# From Risk-Seeking to Risk-Averse: The Development of Economic Risk Preference from Childhood to Adulthood

**DOI:** 10.3389/fpsyg.2012.00313

**Published:** 2012-09-07

**Authors:** David J. Paulsen, Michael L. Platt, Scott A. Huettel, Elizabeth M. Brannon

**Affiliations:** ^1^Department of Psychology and Neuroscience, Duke UniversityDurham, NC, USA; ^2^Center for Cognitive Neuroscience, Duke UniversityDurham, NC, USA; ^3^Center for Interdisciplinary Decision Sciences, Duke UniversityDurham, NC, USA; ^4^Department of Neurobiology, Duke UniversityDurham, NC, USA; ^5^Brain Imaging and Analysis Center, Duke UniversityDurham, NC, USA

**Keywords:** risk, decision-making, gambling, child development, risk preference

## Abstract

Adolescence is often described as a period of heightened risk-taking. Adolescents are notorious for impulsivity, emotional volatility, and risky behaviors such as drinking and driving under the influence of alcohol. By contrast, we found that risk-taking declines linearly from childhood to adulthood when individuals make choices over monetary gambles. Further, with age we found increases in the sensitivity to economic risk, defined as the degree to which a preference for assured monetary gains over a risky payoff depends upon the variability in the risky payoff. These findings indicate that decisions about economic risk may follow a different developmental trajectory than other kinds of risk-taking, and that changes in sensitivity to risk may be a major factor in the development of mature risk aversion.

## Introduction

Imagine you are confronted with a choice between a sure $5 or a coin flip in which you could win $10 or nothing. A rational decision-maker would be indifferent to these options because they have the same expected, or average, value ($5). Yet, most people prefer the sure bet, a phenomenon known as **risk aversion** (Tversky and Kahneman, 1981).

A number of factors have been found to modulate risk aversion. For example, people tend to accept risks more often for smaller than for greater monetary rewards (Weber and Chapman, 2005), more often for primary (juice) than secondary (money) rewards (Hayden and Platt, 2008), and more often when offered multiple opportunities to wager than when offered only a single shot (Redelmeier and Tversky, 1992). The timing of gambles can also be a factor: risk-taking decreases when consecutive choices are spaced at longer temporal intervals (Hayden and Platt, 2007). Some species display similar risk preferences and decision strategies, for example humans and macaques show win-stay and lose-shift strategies for juice rewards (Hayden and Platt, 2008), while others, such as chimpanzees and bonobos, display risk-seeking, and risk-averse tendencies, respectively (Heilbronner et al., 2008).

Age is another factor modulating risk aversion. Several studies have shown that risk aversion increases slowly between childhood and adulthood (Levin and Hart, 2003; Levin et al., 2007; Rakow and Rahim, 2010; Weller et al., 2010). Young children are more influenced than adults by the probability of winning (Harbaugh et al., 2002), and more likely to take economic risks that are disadvantageous in the long run. Nonetheless, they are also less likely to take risks that are advantageous (Crone et al., 2008). Other studies have shown that adolescents are more likely than adults to be predisposed to take risks by changes in affect as well (Figner et al., 2009; Burnett et al., 2010). Thus, while aversion to risk appears to increase monotonically with age, modulating factors like emotional affect and probability can alter this general pattern.

**Figure 1:**
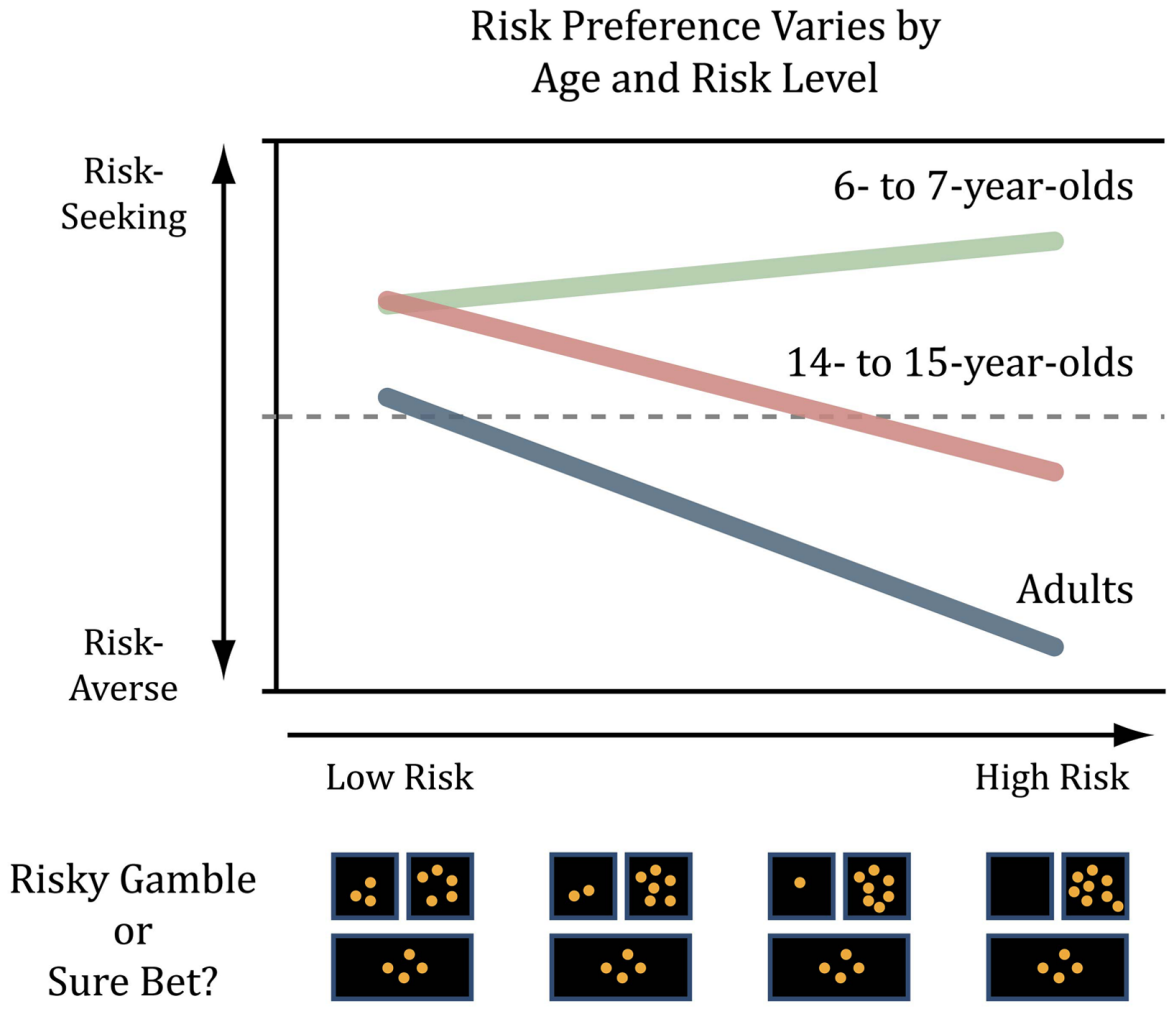
**Children, adolescents, and adults, were presented with the choice between accepting risky gambles at varying levels of risk and taking a sure bet**. The figure shown here illustrates that risk-seeking declined with age, and was modulated by level of risk. With increasing risk, children were risk-seeking, while adults and adolescents were risk-averse.

In a recent article, we reported that risk aversion increases both with age and with increasing economic risk (Paulsen et al., 2011b). We presented children, adolescents, and adults with choices between a sure bet and a risky gamble with equal expected value while varying the disparity between the maximum and minimum rewards available for the gamble. For example, a trial with a low-risk gamble could present the chance of winning either 5 or 3 coins vs. a sure bet of 4 coins, whereas a high-risk gamble could present the chance of winning either 8 or 0 coins. We used the **coefficient of variation (CV)**, mathematically defined as the standard deviation divided by the mean, of the possible outcomes as our measure of economic risk for two reasons. First, because CV is a unitless measure of risk, it permits comparison between reward types and species. Second, CV has been demonstrated to be a better predictor of choice behavior – accounting for greater behavioral variance in risk vs. sure bet choices – in humans and other animals than more traditional measures of risk like variance or standard deviation (Weber et al., 2004). In our study, when CV was low, there were no group differences in risk-taking between children, adolescents, and adults. As CV increased, however, age-related differences in choice emerged: children were mildly risk-seeking, adolescents were mildly risk-averse, and adults were reliably risk-averse (Figure 1; Table 1).

**Table 1:** **Proportion risky choices by age group and coefficient of variation**.

	Coefficient of variation			
	0.35	0.71	1.06	1.41
Child	0.623	0.718	0.770	0.703
Adolescent	0.686	0.705	0.443	0.391
Adult	0.539	0.372	0.173	0.167

## Contributions

Our findings make three important contributions to the growing literature on the development of risky decision-making. First, they underscore the need to distinguish between different types of risk-taking. While economic risk tasks allow experimental control and are frequently used to study risk-taking, there may be fundamental differences between this context and more naturalistic risky decision-making situations such as whether to have unprotected sex, use illegal drugs, or drink and drive. Second, our findings suggest that the development of sensitivity to risk may be a major factor in the development of mature risk aversion. Third, our findings indicate developmental differences in how value is assessed. We next consider each of these factors in turn.

### Real-world vs. economic risk

Although real-world risks *de facto* entail economic risk, a general distinction is often made between behaviors like substance use, extreme sporting, and delinquency, that may have longer lasting and less recoverable real-world consequences than the behaviors elicited by laboratory gaming tasks. These two general types of risk-taking may be distinguished in several ways. One is by domain. For example, decision-research suggests that individual risk preference depends on situational variables, like whether the risk involved is recreational, social, health-related, or financial (Weber et al., 2002). A person who appears risk-seeking in their physical activities may be risk-averse in their investments, although mediating factors like perceived control over a situation or the perceived risk involved may reduce apparent discrepancies to a common risk-attitude (Weber and Milliman, 1997).

A second dimension in which these two types of decisions differ is the heightened emotionality and greater potential for serious consequences in real-word as opposed to laboratory gambling. Real-world decisions can lead to financial success or ruin, physical elation or injury, psychological pleasure or addiction, and social rejection or adulation. A handful of studies have attempted to explicitly manipulate the emotionality of decision-making in laboratory risk-taking tasks for example by providing feedback (Burnett, et al., 2010; Figner, et al., 2009), or by having peers present during decision-making (Gardner and Steinberg, 2005).

A third way to distinguish real-world risk from economic risk involves a distinction between **risk** and **ambiguity** (Knight, 1921; Huettel et al., 2006). To understand this distinction, compare the probability of rolling “6” on a fair die (risk) with the probability of being in an auto accident (ambiguity). In the case of a die, the probability distribution for different outcomes is fully specified, whereas the probability of a car crash is dependent on a multitude of contextual factors. With risk, all possible outcomes and their *exact* probabilities are known prior to making a decision. With ambiguity, such information about outcomes is incomplete: road and weather conditions, the experience of other drivers, the frequency of wildlife crossings, and the physical state of car and driver, can all make unknown contributions to the likelihood of an accident. Thus while many real-world “risk-taking” decisions involve ambiguity, laboratory gambling tasks, including our own, involve risks that are more concretely defined.

Real-world risk-taking shows a different developmental trajectory from economic risk-taking, which could be due to any of a number of reasons. Health statistics show increased mortality in adolescents due to preventable causes like drug abuse and driving under the influence of alcohol (Eaton et al., 2006; Karch et al., 2009), suggesting that real-world risk-taking has a curvilinear, inverted U-shaped pattern from childhood through adolescence and into adulthood. One contributing factor to this pattern could be that adolescents are very sensitive to the influence of their peers (Lewis and Lewis, 1984; Gardner and Steinberg, 2005; Chein et al., 2010). Adolescent delinquency, one form of risk-taking, may reflect attempts to establish adult-like independence (Moffitt, 1993). Another possibility is that adolescents’ heightened subjective estimate of their own control over situational variables may lead them to take greater risks (Weber and Milliman, 1997; Reyna and Farley, 2006).

Some authors have suggested that the amplified emotional volatility and/or mood fluctuations associated with adolescence (Buchanan et al., 1992) may influence decisions in contexts involving risk (Burnett, et al., 2010; Figner, et al., 2009), perhaps due to an affective focus on reward which overshadows attention to losses. Adolescents may also just think less before acting (Steinberg, 2004) or act impulsively (Steinberg et al., 2009). Heightened emotions and impulsivity might also work together to increase risk-taking by promoting action before the potential negative consequences of actions have been fully considered. Endorsing this idea, the neural mechanisms responsible for impulse control take time to mature, after sensitivity to reward has already emerged (Galvan et al., 2006; Casey et al., 2008). Thus, based solely on the findings from research on real-world risk-taking, our results would not have been predicted, showing that not all types of risk are appreciated equally across age, and that the distinction between different kinds of risk is an important one to make.

### Development of risk-sensitivity

Our findings also suggest that **risk-sensitivity** changes across childhood and adolescence. We found that while children prefer greater CVs, adolescents and adults found greater CVs aversive. This pattern of behavior indicates a positive correlation between risk-taking and CV in children, and a negative correlation in adulthood. The transition during development from a positive to a negative slope between risk preference and the CV of a gamble implies a time period when the risk of a gamble has no predictive power on choice behavior. Given the hypothesis of competing processes in risk-taking behavior (Steinberg, 2010) – e.g., impulsivity vs. self-control and reward-seeking vs. loss-aversion – such indifference to risk suggests a period of balance between two or more processes involved in economic risk-taking. Knowing when this period of balance occurs can inform our understanding of the impact of other associated neural and cognitive changes in development (e.g., executive function, temporal discounting, etc.). Ongoing work suggests that the cross-over from risk-seeking to risk-averse behavior occurs between 7.5 and 13 years of age (Paulsen et al., 2012). In addition, this information indicates that children younger than 8-years-old will provide data pivotal to identifying the development of decision-making under risk.

### Valuation and evaluation

Apart from sensitivity to risk, the fact that gambles typically become less attractive as individuals age may also indicate developmental differences in how value is assessed. One possibility is that value is derived from the quantifiable properties of the gamble, like magnitude and probability, or through an interaction between these objective measures and how they are perceived. For example, in Cumulative Prospect Theory (Tversky and Kahneman, 1992), the value of a gamble is formed as the summed product of individually weighted probabilities and individually weighted values. Greater attention to one or another outcome modulates these weightings (Lopes, 1995): focusing more on gains yields an optimistic weighting and hence greater value, while focusing more on losses yields a pessimistic weighting and hence lesser value. Evidence in favor of this effect of attention comes jointly from behavioral differences in children and adult's behavior in gain and loss domains (Weller, et al., 2010; Paulsen et al., 2012) and from eye-tracking studies showing that the amount of time spent looking at a particular option is predictive of its being chosen among alternatives (Krajbich and Rangel, 2011). Thus, it's possible that children and adults differentially allocate attention to the winning and losing outcomes of a gamble, with younger individuals putting more focus on the jackpot, less focus on the loss, or both, compared to adults.

Another factor that may change over development is the inherent preference for the novelty or uncertainty of gambles. Prior research has shown that in monkeys the gamble itself has inherent value (Hayden et al., 2008). Several lines of research have found novelty-seeking to be particularly elevated during adolescence (see Spear, 2000, for review). However, many studies operationalize novelty-seeking as exploration of novel environments, and similar to the distinction between real-world and economic risk-taking, there may be differences between exploration and gambling related novelty-seeking. From this, the value of novel or uncertain outcomes compared to predictable outcomes may have a different developmental trajectory, namely decreasing with age. Consistent with this idea, although there was no significant difference between age groups in the probability of choosing the gamble when it was low-risk, children and adolescents showed an overall preference for the risky option, while adults did not. Future work will be needed to test these hypotheses, and to better separate novelty-seeking, exploration, and risk-taking, from one another.

Much decision-making research has focused on how gambles are appraised, that is to say the valuation stage of decision-making, and less on how the assessment of outcomes relates to future decisions (see Rangel et al., 2008, for discussion of decision-making stages). Age-related differences in outcome evaluation and learning could also contribute to the patterns we observed. For example, regret, an important mediator of decision-making, develops slowly between childhood and adulthood (Habib et al., 2012). Neuroimaging work using the same task we focused on here found that the hippocampus, amygdala, and insula, among other regions, increased in activation with age during decision-making (Paulsen et al., 2011a). Both hippocampal and amygdalar regions contribute to learning and memory, while the insula is thought to integrate affective and cognitive information during decision-making (Preuschoff et al., 2008). Learning from outcomes tends to improve with development (e.g., Crone and van der Molen, 2004). Taken together, these ideas lead to the hypothesis that adults may be more adept at incorporating the outcomes of prior decisions into subsequent decision-making contexts. Future studies will need to explore this hypothesis further.

## Conclusion

The findings in Paulsen et al. (2011b) suggest a more gradual emergence of risk aversion over age compared to the curvilinear patterns often found in real-world risky behaviors. Precisely how risk-taking tasks relate to real-world risk-taking remains an active area of research (Winters and Anderson, 2000; Bruine de Bruin et al., 2007; Morrongiello et al., 2009). Understanding the behavioral and neural changes in decision-making and its neural mechanisms over development could influence the design of interventions that could help to preclude or ameliorate the harmful consequences of risk-taking during development.

## Conflict of Interest Statement

The authors declare that the research was conducted in the absence of any commercial or financial relationships that could be construed as a potential conflict of interest.

## Key Concept

Risk aversion: The tendency to prefer certain over risky options. Risk aversion is most clearly identified when the certain and risky options under consideration have the same average or expected value.
Coefficient of variation (CV): Mathematically defined as the standard deviation of outcome values divided by the mean. In our tasks, CV of outcomes is used as a measure of risk. Note that this definition works for values that are either all positive or all negative.
Risk: Uncertainty when possible outcomes and their probability of occurrence are known. For example, each face of a fair-sided die has a 0.166 probability of landing face-up. Betting on the outcome of a roll is risky.
Ambiguity: Uncertainty when information about possible outcomes and probability is incomplete or missing. For example, a used automobile has high mileage and an unknown history of owners. Expecting this car to last 5-years with only the given information is full of ambiguity.
Risk-sensitivity: Sensitivity to changes in degree of risk, indicated by changes in behavior or preference.
